# Liver microsystems in vitro for drug response

**DOI:** 10.1186/s12929-019-0575-0

**Published:** 2019-10-28

**Authors:** Jyong-Huei Lee, Kuan-Lun Ho, Shih-Kang Fan

**Affiliations:** 1 Department of Mechanical Engineering, National Taiwan University, Taipei, Taiwan; 2 Department of Mechanical and Nuclear Engineering, Kansas State University, Manhattan, KS 66506 USA

**Keywords:** Engineered liver microsystems, Drug response, Cell micropatterning, Hydrogel biofabrication, Microfluidic perfusion

## Abstract

Engineering approaches were adopted for liver microsystems to recapitulate cell arrangements and culture microenvironments in vivo for sensitive, high-throughput and biomimetic drug screening. This review introduces liver microsystems in vitro for drug hepatotoxicity, drug-drug interactions, metabolic function and enzyme induction, based on cell micropatterning, hydrogel biofabrication and microfluidic perfusion. The engineered microsystems provide varied microenvironments for cell culture that feature cell coculture with non-parenchymal cells, in a heterogeneous extracellular matrix and under controllable perfusion. The engineering methods described include cell micropatterning with soft lithography and dielectrophoresis, hydrogel biofabrication with photolithography, micromolding and 3D bioprinting, and microfluidic perfusion with endothelial-like structures and gradient generators. We discuss the major challenges and trends of liver microsystems to study drug response in vitro.

## Introduction

**Fig. 1:**
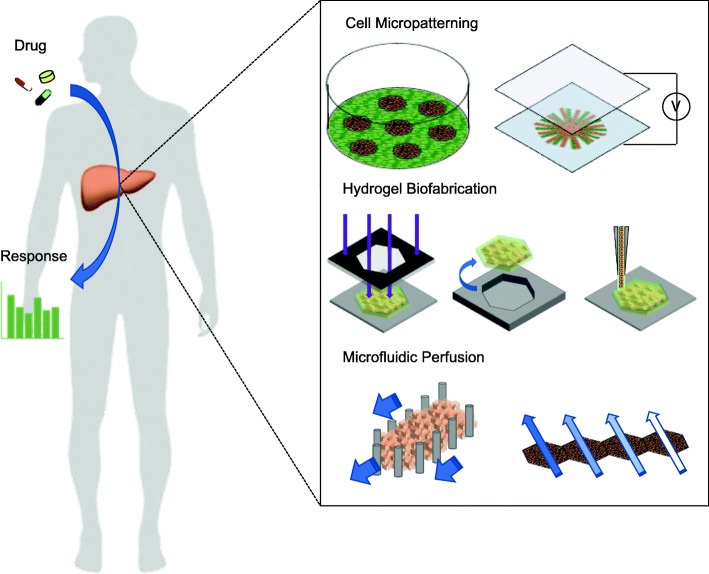
Liver microsystems in vitro for drug responses. Cell micropatterning techniques use soft lithography and dielectrophoresis to arrange precisely the various cells on a micrometer scale. Hydrogel biofabrication techniques apply photolithography, micromolding and 3D bioprinting to reconstruct a 3D heterogeneous extracellular matrix. Microfluidic perfusion culture systems offer endothelial-like structures to mimic flow conditions and gradient generators to reconstruct gradients of oxygen, nutrients and metabolites

Drug development and screening is a costly and lengthy process [1, 2]. To decrease the cost and time, researchers have developed various culture systems in vitro to test drug response. With the advances of microengineering, liver microsystems, or so-called liver-on-a-chip techniques, have demonstrated diverse functions and grown vigorously. The liver microsystems in vitro mimic the conditions in vivo for reliable drug response with cells of minimum number, which relieves the demand for animal testing and decreases the duration before human clinical trials [3]. To create a microenvironment as in vivo for cell culture, various engineering tools have been developed, as shown in Fig. 1. To improve the liver cellular function and to recapitulate the cell arrangements in vivo, cell micropatterning techniques, including soft lithography and dielectrophoresis, have been demonstrated. In addition, hydrogel biofabrication techniques, such as photolithography, micromolding and three-dimensional (3D) bioprinting, provide a heterogeneous engineered extracellular matrix (ECM) that offers a 3D liver tissue to study drug response. Moreover, to reproduce the architectures of liver lobule and sinusoidal, the microfluidic perfusion culture systems use endothelial-like structures to mimic flow conditions and gradient generators to reconstruct gradients of oxygen, nutrients and metabolites. In this review, we introduce and compare several representative engineering methods established for diverse cell sources, hydrogels and bioassays to build liver microsystems in vitro to study drug response.

## Cell micropatterning

**Fig. 2:**
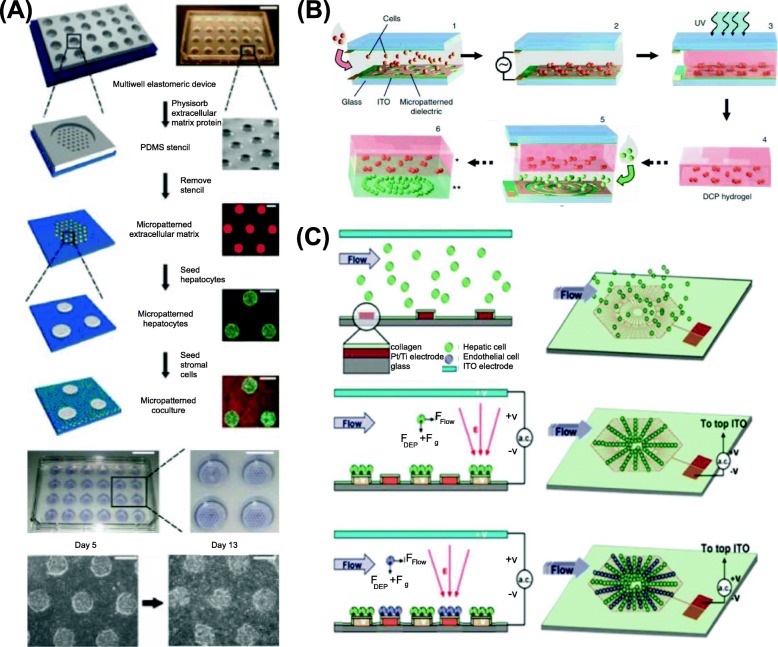
Cell-micropatterning techniques. **a**. Soft-lithography-based coculture microsystem compatible with bioassays on bench and plate readers [5]. **b**. DEP driving primary rat hepatocytes toward regions of large electric field to form cell clusters [12]. **c**. Array of lobule-mimetic-stellate electrodes sequentially constructing a coculture condition with DEP [13]

It is difficult to maintain liver functions of primary hepatocytes in long-term monoculture conditions [4]. To solve this problem, scientists introduced micro-coculture systems with soft lithography adopted from semiconductor fabrication [5–11]. As shown in Fig. 2a, by soft lithography the hepatocytes, selectively attached on the micropatterned collagen, and the supporting stromal cells (fibroblasts) were further seeded to achieve effective two-dimensional (2D) cell coculture. The coculture condition greatly enhanced the secretion of albumin and urea, markers of protein synthesis and nitrogen metabolism in hepatocytes, relative to hepatocyte 2D monoculture and lasted for several weeks [5]. Moreover, with the soft lithography micropatterning technique, the ratio of fibroblasts to hepatocytes can be optimized with precise control of the area of cell adhesion, e.g., hepatocyte islands of diameter 500 μm with spacing 1200 μm center to center [6, 7]. The system is compatible with bioassays and plate readers on a bench; it has been used in tests of drug hepatotoxicity and drug-drug interactions [5]. Mitochondrial activity was evaluated using tetrazolium-(MTT)-based colorimetric assay to obtain the half-maximal inhibitory concentration (IC50) values. Furthermore, the cell micropatterning technique based on soft lithography has already been commercialized [10] and applied in pathogen studies, including hepatitis B viruses, hepatitis C viruses and plasmodium pathogens [11].

Dielectrophoresis (DEP), another microengineeirng technique for cell sorting in a biocompatible hydrogel matrix or in a DEP buffer solution on applying a non-uniform electric field, has been widely investigated [12–15]. As shown in Fig. 2b, according to the design of electrode patterns, the DEP force drove hepatocytes towards regions of large electric field to form cell clusters, which facilitates the adjustment of cell organization within the 3D polyethylene-glycol (PEG) hydrogel [12]. As shown in Fig. 2c, with an appropriate operating procedure, hepatoma G2 (HepG2) and human umbilical-vein endothelial cells (HUVEC) were patterned sequentially onto a lobule-mimetic-stellate-electrode array to construct coculture conditions [13], preserving interactions cell to cell that are crucial for further enzyme induction studies [16]. The last, to provide a reusable platform for patterning cells within a 3D hydrogel and a seamless transfer, HepG2 were patterned within an agar hydrogel supported with a paper substrate, which was subsequently positioned into a 96-well plate for culture and analysis [15]. The electric conductivity of the buffer solution or hydrogel matrix must be adjusted for effective DEP actuation without heating and electrolysis [17]. For example, the conductivity of the DEP buffer solution (e.g., 10 mS/m) is much less than that of a normal cell-culture medium DMEM (Dulbecco’s Modified Eagle Medium, conductivity 1800 mS/m) [17]. The frequency of the DEP driving electric signal is another significant parameter that influences the magnitude and direction of the DEP force based on the Clausius–Mossotti factor [18].

## Hydrogel biofabrication

**Fig. 3:**
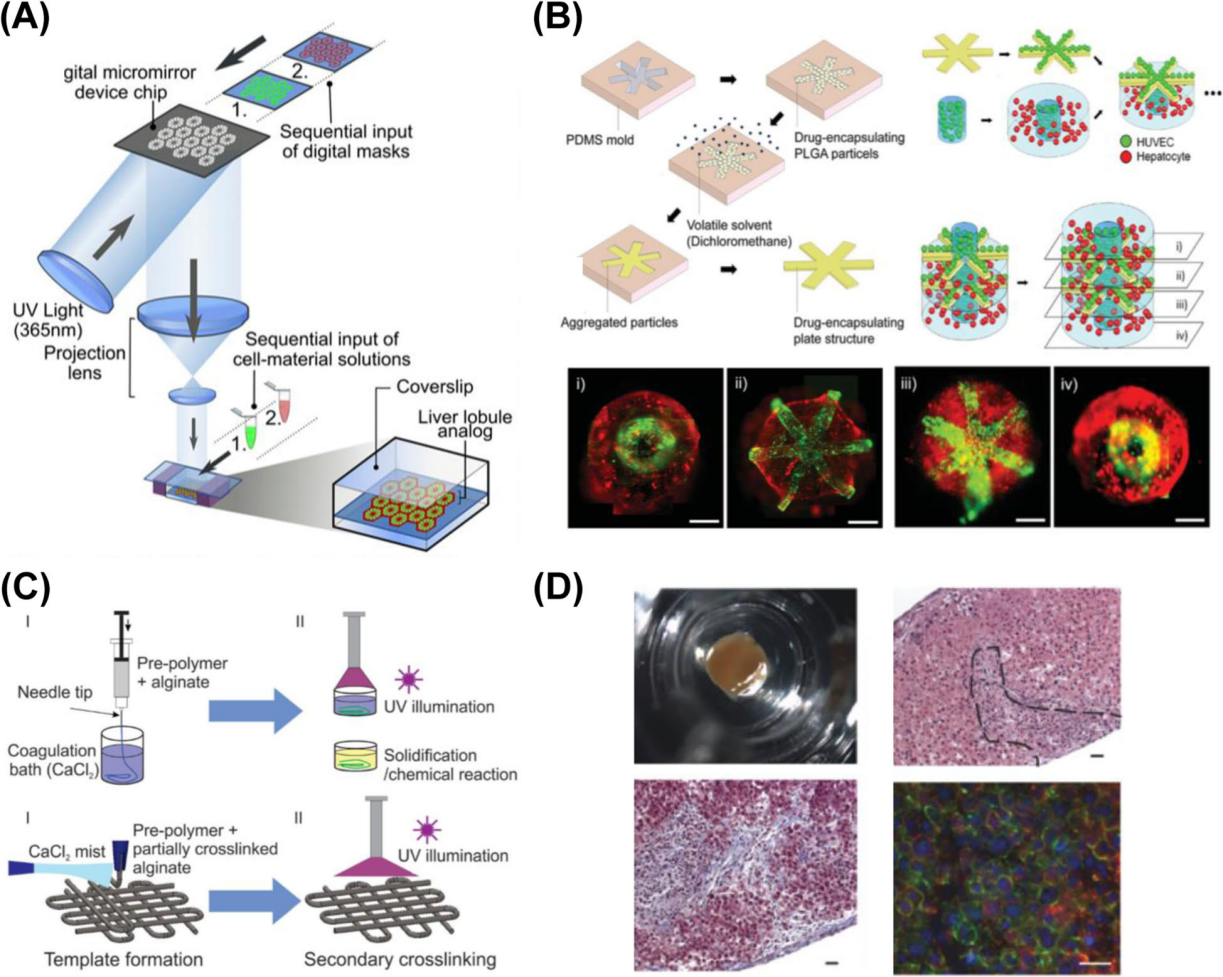
Hydrogel biofabrication of liver tissues. **a.** Photolithographic method constructing heterogeneous structures for cell coculture [21]. **b.** Micromolding patterning drug-encapsulated PLGA particles and cell-encapsulated hydrogels to study cancer therapy [23]. **c.** 3D bioprinting, injecting and curing the biomaterials to form a biomimetic tissue [24]. **d.** 3D liver tissue printed with a commercial 3D bioprinter [25]

From a tissue-engineering point of view, a 3D engineered environment with cells arranged at appropriate positions within an ECM is essential. To obtain such an engineered 3D heterogeneous liver tissue, photolithography, micromolding and 3D bioprinting for a hydrogel, the engineered ECM have been investigated. Inspired by semiconductor fabrication, photolithographic methods have been adopted to transfer the patterns from a mask to the photo-crosslinkable cell-laden hydrogels with UV crosslinking for cell culture [19–22]. The micrometer resolution is sufficient for construction of the cell environment; serial exposures make heterogeneous microstructures obtainable. The mechanical stiffness of a hydrogel can be adjusted with the exposure dosage and the concentration of the hydrogel prepolymer solution. Using digital light processing (DLP) [21], the gelatin methacryloyl (GelMA, 5%) with human induced pluripotent stem cells (hiPSC) and the GelMA (2.5%) with supporting cells were sequentially crosslinked to form a human hepatic lobule structure (Fig. 3a). Compared with a 2D cell monolayer and a 3D hepatocyte-only monoculture, the engineered liver tissue showed greater albumin, urea secretion and enzyme (cytochrome P450) activities after Rifampicin induction [21], which demonstrated the maturation in vitro of hiPSC-derived hepatic cells with liver-specific gene expressions [26]. However, the photolithographic method might be accompanied by some damage to cells caused by UV radiation and free radicals generated by the photoinitiator [27].

Micromolding is another way to pattern hydrogels on a microscale. Unlike photolithographic methods, micromolding is suitable for thermally and chemically crosslinkable hydrogels to avoid UV exposure damage [23, 28–30]. The drug-encapsulating poly (lactide-co-glycolide) (PLGA) particles patterned with micromolding were used for cancer studies [23] (Fig. 3b). As shown in the experimental results, the agents for the anti-vascular endothelial growth factor (anti-VEGF) enhanced the efficacy of chemotherapy on inhibiting the growth of endothelial cells, demonstrating a platform in vitro near that of clinical data [31]. By micromolding varied hydrogels embedded with cells and drugs, the method developed a tumor model in vitro for tests of cancer-therapy drug response.

3D printing (additive manufacturing technique) has been applied to biological and medical fields for its great flexibility; various 3D bioprinters are available on the market with diverse tissues printed [32, 33]. 3D bioprinting injects and cures the biomaterials to form a biomimetic tissue [34] and even an organ, including printed liver tissues to assess responses to clinical drugs [24, 25, 35–41] (Fig. 3c). As shown in Fig. 3d, a 3D printed liver tissue was used to test Trovafloxacin (antibiotic with hepatotoxicity) [25]. The 3D bioprinter can print scaffold-free liver tissue, which is composed of hepatocyte spheroid without any engineered ECM [37]. Another feature of 3D bioprinting is the core-shell structure constructed by a coaxial nozzle [39]. By the coaxial nozzle, the tissue can be printed with a shell for mechanically supporting and a suitable core for liver cell growth [40, 41]. Significant decrease of both albumin secretion and ATP production of the 3D printed liver tissue was observed at doses that induced no hepatotoxicity in standard 2D culture conditions [42], showing that the appropriately printed 3D liver tissues exhibited a greater sensitivity to drug toxicity than the 2D cultured cells [43]. However, the pressure and shear stress at the dispensing nozzle during the printing might cause harm [44]. For example, when the shear stress increased beyond 150 kPa (~ 21.8 psi), the cell viability through a bioprinting nozzle (250 μm) decreased to less than 50%. In general, using 150-μm nozzles, the acceptable dispensing pressure should be less than 10 psi [44, 45]. Although using smaller pressure or a larger nozzle decreases the shear force, the printing speed and resolution are sacrificed. Printing cells with the required resolution with minimum cell damage is hence a critical issue.

## Microfluidic perfusion

**Fig. 4:**
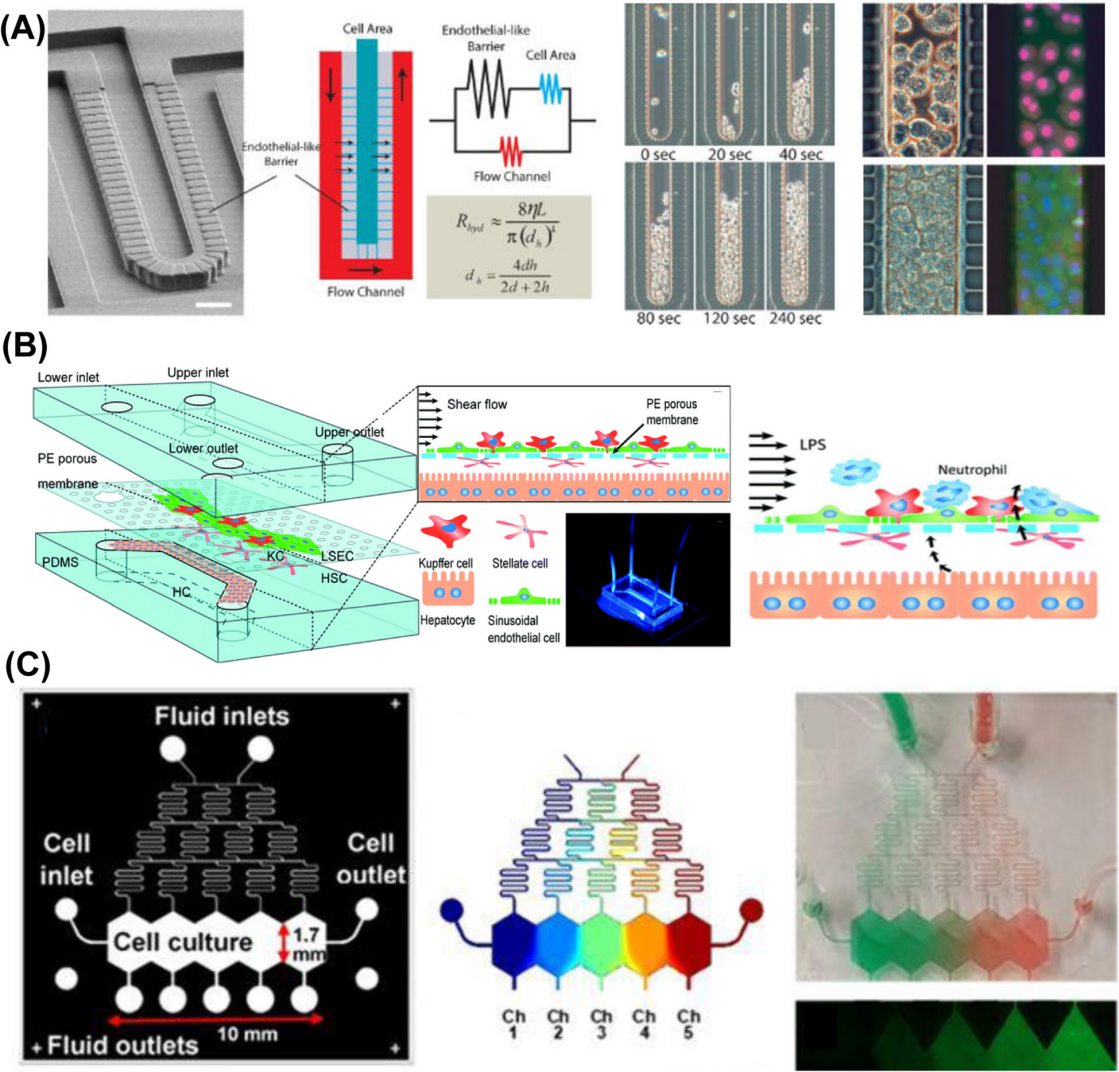
Microfluidic perfusion culture systems. **a.** Artificial endothelial-like structures mimicking the microenvironment in vivo to retain phenotypes and functions of primary hepatocytes [46]. **b.** Complicated model of immune response of neutrophil recruitment [57]. **c.** Microfluidic gradient generator to study liver zonation [59]

Although static cell cultures are widely favored in many biological laboratories, a system for microfluidic perfusion culture provides a more biomimetic situation [46–61]. Microfluidic-based microsystems generate flow conditions as in vivo for perfusion cell culture with decreased sample usage and realize a dynamic cell culture with a continuous transfer of nutrition and metabolites. The liver sinusoidal endothelial fenestrations are special differentiations for substance exchange and protection of the hepatocytes from the shear flow of blood [62]. The artificial endothelial-like structures, made of polydimethylsiloxane (PDMS) via micromolding, reproduced the flow rates in vivo (Fig. 4a) [46] (e.g., 10 nL/min in the transport channel and 0.007 nL/min in endothelial-like structures), which retained the phenotypes and functions of primary hepatocytes [46–48] and even formed bile canaliculi [49]. The microfluidic system pumped and regulated various drugs of varied concentration on a single chip, which facilitated drug screening. The IC50 values evaluated from the microfluidic chip correlated with the reported median lethal dose (LD50) values in rat experiments [48]. The microfluidic systems also promoted the differentiation efficiency of stem cells to hepatic or hepatocyte-like cells [55, 56].

Moreover, a microfluidic-based microsystem is suitable for coculture studies. With a porous membrane, microfluidic systems mimicked complicated multiple cell–cell interactions of liver sinusoidal structures [57]. The coculture with non-parenchymal cells of three kinds -- liver sinusoidal endothelial cell, Kupffer cell, hepatic stellate cell -- in a calculated shear flow (shear stress 0.1–0.5 dyn/cm^2^) environment enhanced albumin secretion and cytochrome P450 (CYP) enzyme activities. Stimulated by the lipopolysaccharide and neutrophil recruitment, the microfluidic system demonstrated an immune response of neutrophil adherence as a prospective drug screening platform (Fig. 4b).

Another advantage of a microfluidic system is the ability to provide a stable gradient for liver zonation as in vivo. The liver zonation is a spatial gradient of oxygen, glucose, albumin, urea and other metabolites caused by the circulation of blood. Zone 1 is rich in oxygen and nutrients, and has higher cell metabolic functions and stronger regenerative capacities, whereas the conditions of hepatocytes in zone 3 are poor and the cell regeneration ability is also weak; the hepatocytes therein are susceptible to drugs and toxic substances. The microfluidic gradient generator [59] established zonation of carbohydrate, glucose, nitrogen and xenobiotic metabolism to build a liver metabolic zonation model for zonal drug toxicity response (Fig. 4c). In this study 3-methylcholanthrene (3-MC) to induce CYP1A enzymes activities [63] was used at varied levels with a gradient generator of concentration (0–2 μM within distance 10 mm) and exposed under acetaminophen (a medicine for pain relief that has hepatotoxicity in an excessive dose [64]) to generate cell toxicity.

The drug metabolism and pharmacokinetics are pivotal points when developing new drugs. For the whole-body drug metabolism and pharmacokinetics analysis, microfluidics is the most suitable platform because it can systematically integrate multiple organs on one single chip [65, 66]. The Gut-liver microfluidic chip is developed for drug metabolism and pharmacokinetics research. The apigenin is used as the model drug and the coculture model has a higher metabolic rate than monoculture model, which is similar to animal experiments [67]. In addition, the small intestine–liver-lung microfluidic chip is used for testing three kinds of anticancer drugs (epirubicine, irinotecan, and cyclophosphamide). The anticancer drugs act on the target cells shows that this platform can replicate the in vivo pharmacokinetic [68]. Besides that, the liver-kidney microfluidic chip is applied to study hepatotoxicity and nephrotoxicity of drug metabolites [69, 70]. The microfluidic provides a drug screening platform for multiple organs.

## Comparisons

Table 1 compares the engineering methods, corresponding drug-response studies, advantages and disadvantages to achieve liver microsystems in vitro. As liver is the main detoxifying organ in a human body, the drug hepatotoxicity is important and can be studied with live microsystems in vitro. To evaluate the toxicities at varied drug concentration, cell viability, albumin secretion and IC50 are common factors to quantify hepatotoxicity. For various purposes, such as drug hepatotoxicity, drug-drug interactions, metabolic function and enzyme induction, various drugs were applied.

**Table 1:** Summary of liver microsystems in vitro

Engineering method	Drug response study	Advantage	Disadvantage	Hepatocyte type	Engineered ECM
Soft lithography [5–11]	- Drug hepatotoxicity [5, 9] - Drug-drug interactions [5] - Enzyme induction [5]	- High cell interaction between different cells in 2D coculture	- Low relevance to liver lobule anatomy - Lack of 3D morphology	- Primary cell [5–8, 10, 11] - Cell line [9]	- Collagen [5–11]
DEP cell patterning [12–15]	- Enzyme induction [13]	- Direct cell patterning	- Buffer or hydrogel with small conductivity	- Cell line [12–15]	- Agarose [12, 15] - Collagen [13] - PEG hydrogel [12]
Hydrogel photolithography [19–22]	- Enzyme induction [21]	- Patterning heterogeneous biomaterials - High resolution	- Damages by UV radiation and free radical	- Primary cell [20] - Stem cell [21] - Cell line [19, 22]	- Gelatin [22] - GelMA [19, 21] - PEG hydrogel [19] - PLA [20]
Hydrogel micromolding [23, 28–30]	- Cancer therapy [23] - Drug hepatotoxicity [28] - Metabolic function [29]	- Patterning heterogeneous biomaterial	- Poor flexibility to complicated geometry	- Primary cell [30] - Stem cell [29] - Cell line [23, 28]	- Fibrin gel [30] - GelMA [28] - PEG hydrogel [23] - PLGA [23] - POMaC [29]
3D bioprinting [24, 25, 35–41]	- Drug hepatotoxicity [25] - Enzyme induction [25, 36, 37] - Transplantation [41]	- Patterning heterogeneous biomaterial - Directly printing biomaterial in 3D space - Large-scale printing	- Large pressure and shear stress during the printing	- Primary cell [24, 25, 37, 41] - Stem cell [38] - Cell line [24, 36, 40]	- Alginate [40] - Collagen [24, 40] - Gelatin [36] - GelMA [24, 40] - Matrigel [40, 41] - NovoGel [25] - PEG hydrogel [40]
Microfluidics [40–55]	- Drug hepatotoxicity [46, 48, 51, 52, 58, 59, 61, 68–70] - Drug metabolism and pharmacokinetics [67–70] - Drug-drug interactions [52, 59, 60] - Enzyme induction [48, 53, 54, 59, 60, 67, 69] - Liver immune [53, 56, 60] - Liver zonation [36, 50, 59]	- Perfusion culture as in vivo - Automation - Small sample volume - Gradient generator	- Closed culture environment	- Primary cell [46–49, 53, 56–61] - Stem cell [54–56] - Cell line [47, 50–52, 55, 56, 60, 67–70]	- Agarose [51] - Collagen [48–53, 56, 59, 68–70] - Fibronectin [67] - Gelatin [51] - PEGDA [54]

In addition, the level of alanine aminotransferase (ALT) and aspartate aminotransferase (AST) in serum are also indicators of liver damages and the ratio of AST/ALT is useful in the diagnosis of liver disease [71, 72]. For the microsystem, the AST level in the cell culture medium is measured to evaluate cell injury level [70]. Although the use of ALT or AST as an indicator of liver damage is rare in the field of the liver microsystem, it is still an important way to evaluate hepatitis. As the main organ for drug metabolism, liver plays a crucial role in eliminating many therapeutic drugs. Among the most important drug-metabolizing enzymes is cytochrome 450, a family of enzymes that function as monooxygenases, which are mostly found in the liver [73]. Some of the in vitro live microsystems have demonstrated better enzyme expression or metabolic activities compared to conventional methods [5, 13, 21, 25, 36, 48, 53].

The cells and hydrogels used in the engineering methods are also highlighted. The liver is composed of orderly-aligned hepatocytes and non-parenchymal cells within ECM. Hydrogels, such as collagen [5–11, 13, 35], agarose [12, 15], PEG [12, 19, 23] and GelMA [19, 21, 24, 28], are widely used in liver microsystems as the engineered ECM [74–76] to support the initial growth of cells. In studies of drug response, the source of hepatocytes and the cell types of non-parenchymal cells are crucial [75, 77, 78]. Through the progress of biotechnology, the hepatocytes can be obtained from isolation of human or animal liver cell, stem-cell differentiation and cell line development [2, 3, 79, 80]. For the preclinical research on drugs, the primary cells isolated from a human being or an animal have greater physiological relevance and retain a high level of enzyme activity, phenotype and function [2, 3], but the primary hepatocytes are difficult to obtain and to maintain liver function during long-term culture [2, 3]. Coculture with fibroblasts or other stromal cells is hence widely adopted for long-term culture of primary hepatocytes [5–8, 10, 11, 20, 30, 35]. Hepatocyte derived from stem cells offers a patient-specific cell source for research on liver drug response in vitro [81, 82], but the differentiation and culture of stem cells is more challenging [83]. Despite a low sensitivity to drugs and loss of some phenotypes, cell lines derived from liver tumors are commonly used in an early stage of microsystem development [12–15, 19, 22–24, 28, 36] for the accessibility and capability of multiple passages [84–86].

## Conclusions and future trends

We summarize the possibilities and the limitations of liver microsystems in vitro based on engineering methods of cell micropatterning, hydrogel biofabrication and microfluidic perfusion. As mentioned above, the cell-micropatterning techniques focus on patterning cells on a scale of a few micrometers and hydrogel biofabrication focuses on biomaterial patterns on a scale of tens or hundreds of micrometers. Soft lithography is compatible with traditional on-bench bioassays and has been used to test many drugs and even as foreign pathogen models. However, the 2D cell culture has a cell morphology different from conditions in vivo; the usage of fibroblasts is not physiologically identical with non-parenchymal cell types [75]. As for DEP patterning, a non-uniform electric field can pattern cells with a resolution of a few micrometers, but the critical conductivity of the environmental liquid limits its applications. The micropatterned coculture microsystems of hepatocytes are well established, but there are still limitations on forming biomimetic tissues [76].

Hydrogel biofabrication, such as photolithography, micromolding and 3D bioprinting, provides appropriate 3D heterogeneous biomaterial architectures for the corresponding cell types. The 3D cell culture is, in general, more physiologically related to conditions in vivo than 2D cell culture [75]. Photolithography has limitations on material selectivity and UV damage [27], but it can achieve a patterning scale smaller than micromolding and 3D bioprinting [87]. Micromolding can achieve a complicated architecture on stacking the building blocks with diverse geometry [88], but it is less flexible than 3D bioprinting that can directly print a biomaterial in a 3D space. The main challenge of 3D bioprinting liver tissue is that the hepatocyte must bear the pressure and shear stress during the printing [44, 45]. Although a small pressure or big nozzle might be used, the printing speed or the resolution is sacrificed.

The major advantage of microfluidic perfusion culture systems to study liver drug response is the continuous-flow culture environment. To protect the hepatocyte from the flow shear force and to provide a perfused cultured environment as in vivo, the pillar structures and the porous membranes made with polymers are used to mimic the endothelium function, which helps to retain the phenotype and function of the primary hepatocyte and even to form bile canaliculi. With the designed microchannels and automation, a microfluidic system can simultaneously handle drugs of multiple types with varied concentrations, which can realize high-throughput drug screening with a small sample and drug volume. Using primary hepatocytes as the cell source, it can decrease the cell amounts and increase the efficiency of drug screening, which has a great potential to realize personal precision medicine. For the reconstruction of liver zonation in vitro, the gradient generator is facilitated to create a nutrition and metabolic gradient, which is a physiological model that can clarify the zonal drug metabolism.

In sum, we need a powerful tool that can pattern biomaterials and cells on various scales in 3D and can perform drug testing with fluid control on a microscale. With its ability to build complicated tissue and precise fluid control with great flexibility, a multifunctional microsystem might be a solution of next-generation liver microsystems in vitro to study drug response.

## Data Availability

Not applicable.
